# Unique pathological features and drug resistance patterns in cutaneous tuberculosis

**DOI:** 10.3389/fmicb.2025.1574051

**Published:** 2025-05-30

**Authors:** Yuejiao Liu, Runrui Wu, Junjun Liu, Jinlong Dai, Dongxu Liu, Yadong Liu, Yumei Liang, Wen Chen

**Affiliations:** Chinese PLA Key Laboratory of Tuberculosis, Department of Pathology, The 8th Medical Center, Chinese PLA General Hospital, Beijing, China

**Keywords:** cutaneous tuberculosis, pathological features, drug resistance, granulomatous inflammation, macrophage

## Abstract

Cutaneous tuberculosis (CTB), a rare manifestation of extrapulmonary tuberculosis, often presents diagnostic challenges in clinical settings due to its atypical presentation. The definitive diagnosis relies heavily on pathological evaluation, which underscores the importance of understanding the distinct pathological characteristics and drug resistance patterns of CTB, a subject that has not been extensively explored previously. In this study, we conducted a comparative analysis of 59 CTB samples and 59 pulmonary tuberculosis samples, focusing on their clinicopathological features. Our findings reveal that CTB can be characterized by subcutaneous irregular hypoechoic regions on ultrasound, localized soft tissue swelling, and flaky low-density shadows on CT scans, with MRI effectively determining the extent of bone and soft tissue involvement. The two groups had no statistical difference in the positivity rate for acid-fast staining and molecular detection. Notably, the incidence of granulomatous lesions was higher in CTB compared to pulmonary tuberculosis, and the high number of macrophages in the skin may be an important reason. However, other parameters such as caseous necrosis, coagulative necrosis, inflammatory necrosis, acute inflammation, hemorrhage, fibroplasia, and exudation showed no significant differences between the two groups. Intriguingly, many significant differences in drug resistance patterns were found between the CTB group and the control group. But when comparing the secondary CTB group to the control group, the only significant difference found was in resistance to RFP + INH + STR. Overall, our study highlights unique pathological features and drug resistance profiles in CTB, providing valuable insights for more accurate clinical diagnosis and tailored therapeutic strategies.

## 1 Introduction

Tuberculosis (TB), primarily caused by *Mycobacterium tuberculosis* (*M. tuberculosis*), predominantly affects the lungs. However, the bacterium can also invade other organs, leading to extrapulmonary tuberculosis (EPTB) (Rodriguez-Takeuchi et al., 2019). Cutaneous tuberculosis (CTB) is notably rare, comprising only 1–1.5% of EPTB cases. CTB is typically acquired through hematogenous or lymphatic spread or direct contact. Its clinical presentation is influenced by factors such as the infecting strain’s load and pathogenicity, the route of infection, and the host’s immune status (Hanumanthu et al., 2023; Lopes and Pereira, 2023). Initial CTB lesions are often non-specific, manifesting as subcutaneous nodules, inflammation, ulcers, bleeding, and exudation. This non-specificity poses a diagnostic challenge, making CTB difficult to differentiate from bacterial infections, fungal infections, sarcoidosis, or tumors (Mei et al., 2023; Veenstra et al., 2023). The rarity of CTB further exacerbates the likelihood of misdiagnosis or delayed diagnosis in clinical practice, potentially leading to severe patient outcomes (Parajuli et al., 2023). Therefore, enhancing the accuracy of CTB diagnosis is of paramount clinical importance.

The conventional diagnostic approach for TB includes patient history evaluation, clinical symptom assessment, laboratory testing, and imaging studies. In suspected CTB cases, a battery of tests, such as tuberculin skin tests (TST), are employed. However, TST results are influenced by various factors, such as age, immune status, and BCG vaccination, and it cannot distinguish between tuberculosis infection and immune response after BCG vaccination. Pathological examination, including hematoxylin and eosin (H&E) staining, acid-fast staining, and molecular testing, remains the definitive diagnostic method. The hallmark pathological feature of TB is chronic granulomatous inflammation with caseous necrosis. In CTB, ulcers tend to persist longer than in pulmonary tuberculosis (PTB) and are frequently complicated by co-infections, adding to the complexity of the pathological profile (Ahmed et al., 2023; Verma et al., 2023). Prior to biopsy, patients often receive extensive local or systemic treatment, which can introduce additional non-specific pathological changes (Banvolgyi et al., 2023; Lino et al., 2023). Moreover, the skin’s unique immune microenvironment, rich in Langerhans cells and lymphocytes, contributes to further pathological distinctions between cutaneous and pulmonary TB.

The management of cutaneous tuberculosis (CTB) necessitates a comprehensive and consistent approach to anti-tuberculosis medication, adhering strictly to the principles of early, appropriate, regular, and throughout the treatment process (Czech et al., 2022; Kozinska et al., 2023). For patients presenting with mild skin involvement, anti-TB pharmacotherapy alone is often sufficient to achieve recovery. In cases where skin lesions significantly affect the appearance or function of critical areas such as the face or hands, surgical intervention may be considered for lesion removal and reparative procedures (Hock Gin et al., 2022). However, it is crucial to recognize that surgery serves merely as an adjunct to medical therapy. The tuberculosis bacillus cannot be entirely eradicated through surgical means alone, necessitating the use of anti-TB drugs both pre- and post-operatively.

The first-line anti-TB medications commonly employed in clinical practice include rifampicin (RFP), isoniazid (INH), pyrazinamide (PZA), streptomycin (STR), and ethambutol (EMB). The recently released endTB study results showed the non-inferiority of three fully oral, short-course regimens (BLMZ, BCLLfxZ, and BDLLfxZ) in the treatment of rifampin-resistant tuberculosis, all of which include PZA (Guglielmetti et al., 2025). While the overall incidence of tuberculosis has been declining annually, there has been a notable increase in the prevalence of drug-resistant and multidrug-resistant TB strains. This trend underscores the need for a deep understanding of the drug resistance patterns in TB to tailor personalized treatment plans effectively and improve patient outcomes (Takahashi et al., 2023). Skin samples, being more accessible and less invasive compared to samples from lungs or other vital organs, offer a unique opportunity for study. However, the literature lacks comprehensive reports on whether the drug resistance characteristics of CTB align with those of pulmonary tuberculosis, and if these findings can guide the treatment of tuberculosis more broadly. In our study, we have observed the clinical, pathological, and drug resistance features of CTB, analyzing the distinctions between CTB and pulmonary tuberculosis. These insights provide a crucial foundation for this complex disease’s clinical diagnosis and individualized treatment.

## 2 Materials and methods

### 2.1 Clinical sample collection

This study included patients admitted to the 8th Medical Center of Chinese PLA General Hospital from January 2012 to June 2023. The inclusion criteria: age over 18 years, clinical and pathological diagnosis of *M. tuberculosis* infection. Clinically, some diseases, such as non-tuberculous mycobacteria (NTM), leprosy, and sarcoidosis, can mimic the pathological characteristics and clinical manifestations of tuberculosis. In this study, cases with positive molecular detection of *M. tuberculosis* by PCR were included. Patients were excluded if they had malignant tumors, HIV or other immunodeficiency diseases, autoimmune diseases, severe fungal and bacterial infections, or hematologic malignancies. It is mainly because these diseases can greatly affect the pathological characteristics of tuberculosis and immune status. A total of 59 patients with CTB met these criteria. Among them, 33 patients had a history of tuberculosis at other sites, with secondary to pulmonary tuberculosis (16 cases) and pleural tuberculosis (7 cases) being the most prevalent. The remaining 26 cases were primarily cutaneous, with no tuberculosis at other sites. For the control group, 59 patients hospitalized during the same period with a pathological diagnosis of pulmonary tuberculosis (PTB) were selected.

### 2.2 Hematoxylin and eosin staining

A 3 μm thick tissue section was cut from the sample. The section was baked at 72°C for 30 min, followed by sequential xylene treatment (10 min, twice) and dehydration in a graded ethanol series (100, 90, and 80%; 5 min each). After washing, the section was stained with hematoxylin for 2 min, differentiated with 1% hydrochloric acid in ethanol, and washed again. The section was then blued with ammonia water, washed, and stained with eosin for 5 s. Following another wash, the section underwent a dehydration process in graded ethanol (75, 85, 95, 100%), cleared in xylene, and finally mounted with neutral resin.

### 2.3 Acid-fast staining method

Three 3 μm thick tissue sections were prepared from the sample. After preparation, these sections were processed similarly to the H&E staining method up to the washing post-ethanol dehydration. Next, 2–3 drops of carbolic acid red dyeing solution were applied at room temperature for 2–3 h. Decolorization was achieved using 1% hydrochloric acid in alcohol until a light pink color was attained, followed by a 30-s counterstain with hematoxylin. After subsequent washing and differentiation steps with 1% hydrochloric alcohol and ammonia blue reversion, the sections were dehydrated through a graded ethanol series, cleared in xylene, and sealed with neutral resin.

### 2.4 *Mycobacterium* strain identification

**Figure 1:**
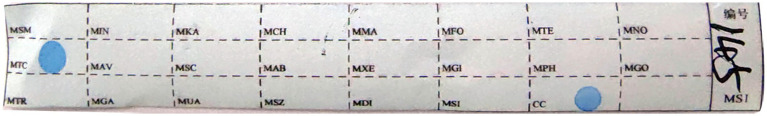
The sequence of the detection site on the membrane strip.

For strain identification, 8–10 tissue slices of 5–10 μm thickness were prepared from each sample and placed into 1.5 mL centrifuge tubes. The procedure involved dewaxing, cracking, digestion, and DNA extraction. In the PCR process, using the *Mycobacterium* species identification gene test kit (Yaneng Biotechnology Co., Ltd., Shenzhen, China), which has obtained a medical device certificate and has been used in our department for over 10 years. Four microliters of sample DNA was added to the PCR tube. Amplification followed a specific protocol: 2 min at 50°C, 10 min at 95°C, then 30 cycles of 45 s at 95°C and 60 s at 68°C, and a final extension of 10 min at 68°C. Hybridization involved placing the membrane strip and amplified product in a test tube with 5–6 mL of Solution A, boiling for 10 min, and then incubating at 59°C for 1.5 h. After washing with Solution B and incubating with a POD enzyme solution, the strips were developed in a color solution, shielded from light for 10 min, and terminated with purified or deionized water. The appearance of blue spots indicated detection, with both positive and negative controls included in each experiment (Figure 1).

### 2.5 *Mycobacterium tuberculosis* drug resistance

Drug resistance testing used the *M. tuberculosis* drug resistance mutation gene detection kit (Yaneng Biotechnology Co., Ltd., Shenzhen, China). Here, 4 μL of DNA was added to each PCR tube. The amplification process was followed by a similar hybridization and washing protocol as described for strain identification. Blue spots on the strips indicated the detection of drug resistance, and each test included positive and negative controls (Li et al., 2022).

### 2.6 Definition of drug resistance type

**Figure 2:**
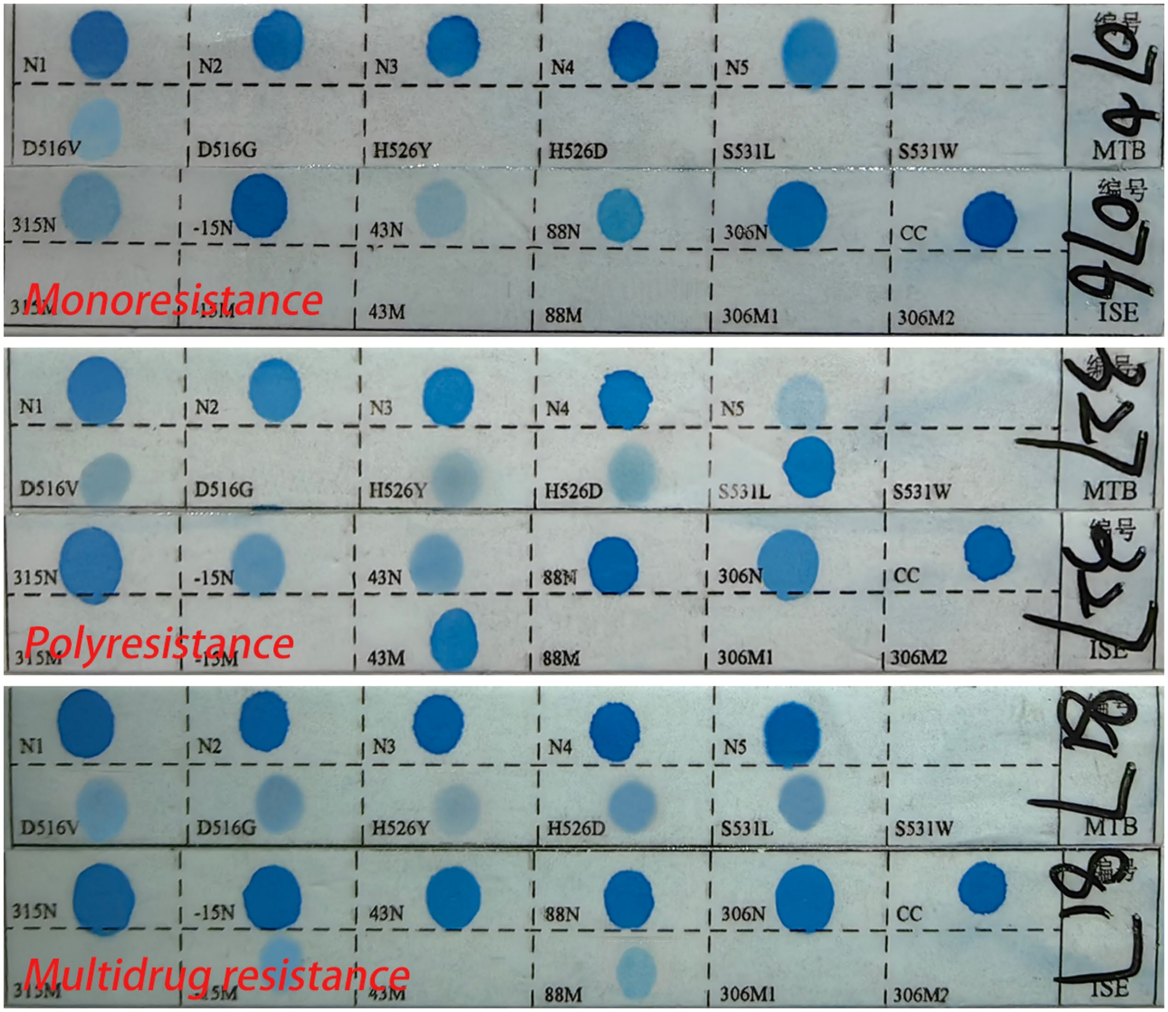
The sequence of drug resistance type on the membrane strip.

Monoresistance refers to resistance to only one antituberculosis drug (Van Rie et al., 2000). Polyresistance refers to resistance to more than one antituberculosis drug but does not include resistance to RFP and INH (Mizukoshi et al., 2021). Multidrug resistance refers to resistance to at least RFP and INH simultaneously (Van Rie et al., 2000; World Health Organization, 2024). Drug resistance to any drug refers to resistance to any one or more antituberculosis drugs (Figure 2). No expression of drug resistance refers to the absence of detectable drug resistance genes, possibly due to low bacterial load.

### 2.7 Statistical methods

Data analysis was conducted using GraphPad Prism 5. Categorical data were presented as frequency (number of cases or strains) and proportion (percentage). The comparison among different groups was performed using the chi-square test or Fisher’s exact test, as appropriate. A *p*-value of less than 0.05 was considered indicative of statistical significance.

## 3 Results

### 3.1 Clinical data

**Figure 3:**
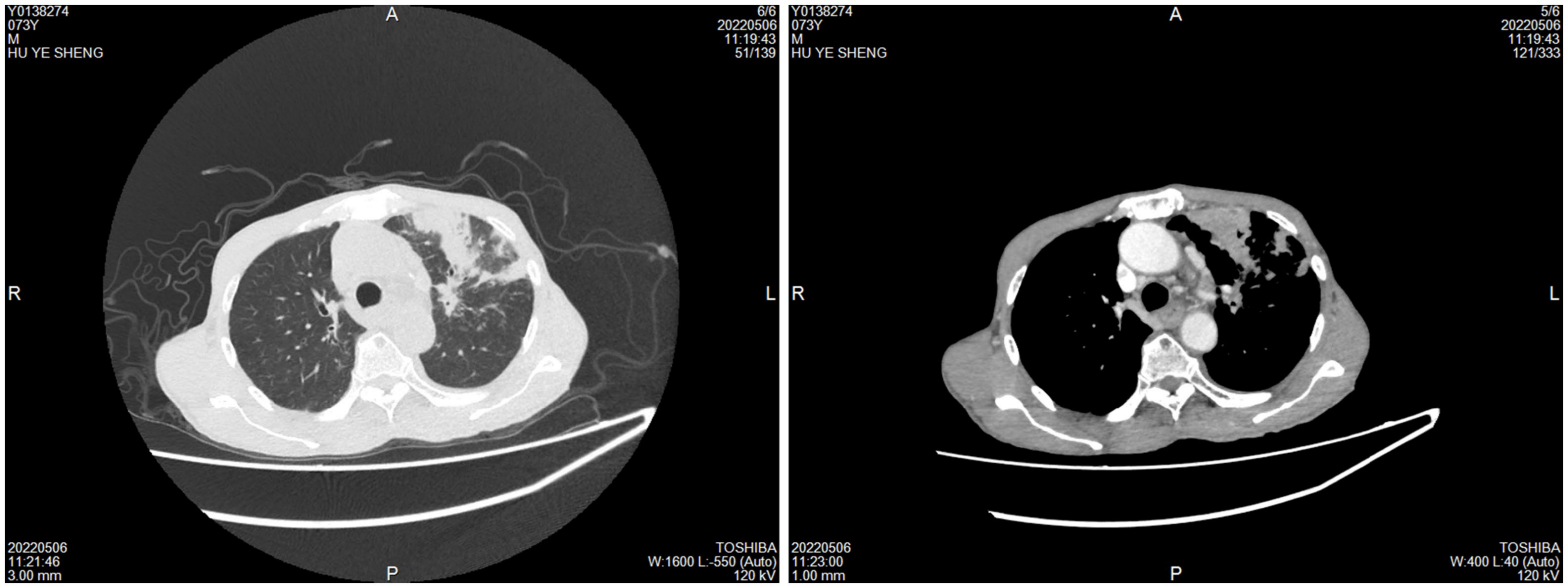
Imaging examination of pulmonary tuberculosis. CT scans revealed lobular and nodular high-density shadows, with cavity shadows observed in some lesions.

The control group comprised 59 pulmonary tuberculosis (PTB) patients, ranging in age from 19 to 78 years, with an average age of 51.78 years. This group included 34 males and 25 females. The CTB group also had 59 patients, aged between 19 and 88 years, with an average age of 45.71 years, including 37 males and 22 females. For the control group, samples were obtained via surgical excision or lung tissue puncture. CT scans revealed lobular and nodular high-density shadows, with cavity shadows observed in some lesions (Figure 3).

**Figure 4:**
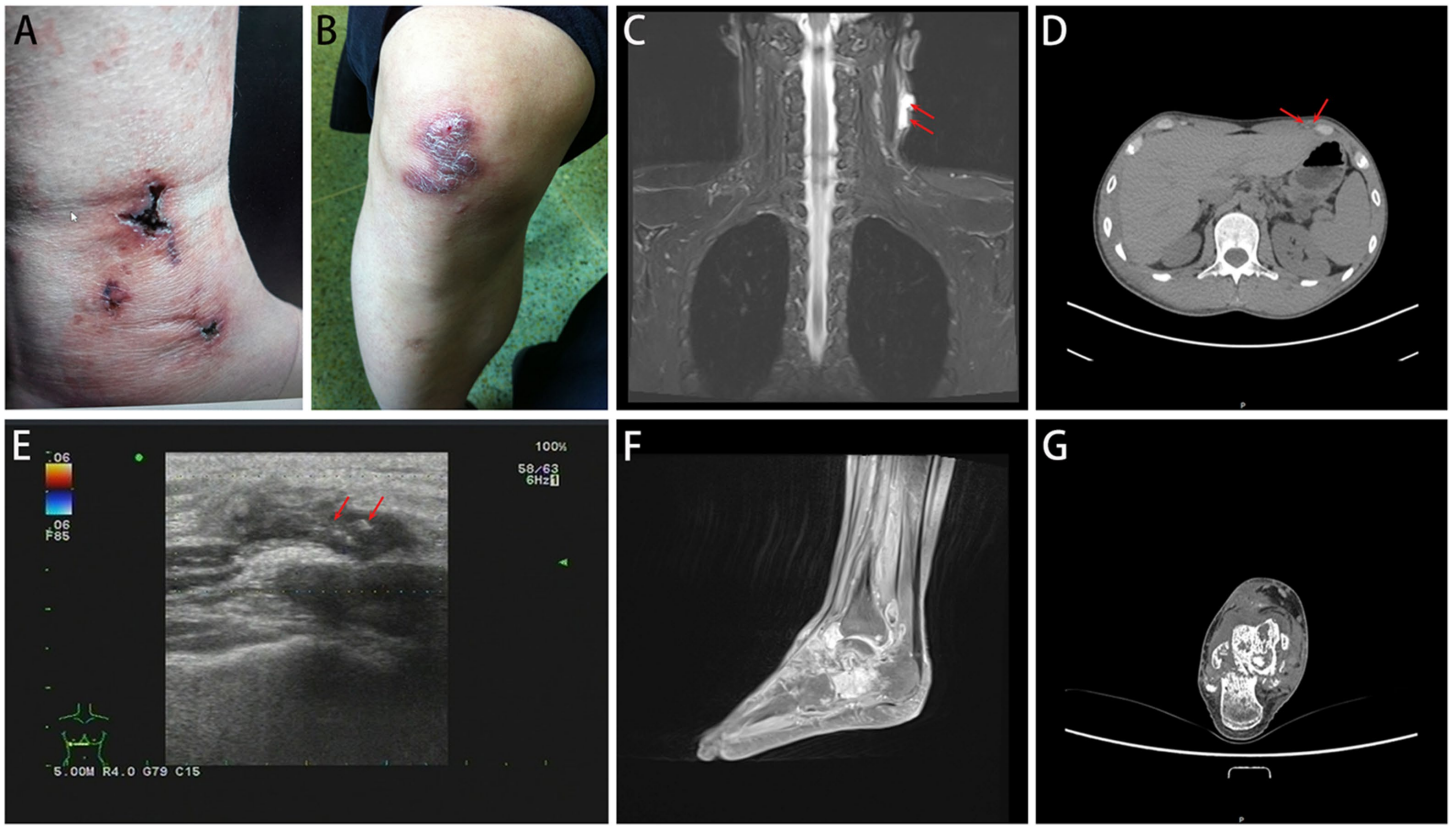
Imaging examination of CTB. CTB appears as ulcers **(A)** or nodules **(B)**. **(C,D)** CT scans indicated local soft tissue swelling and flaky low-density shadows. **(E)** Ultrasound typically showed irregular hypoechoic areas under the skin. **(F,G)** MRI revealed multiple changes in bone and soft tissue.

In the CTB group, the majority (52.54%) of samples were obtained through surgical excision, with 18 cases showing ulcer formation (Figures 4A,B). The sites of infection were predominantly on the trunk (64.41%), limbs (27.12%), and face and neck (8.47%). Ultrasound in CTB cases typically showed irregular hypoechoic areas under the skin, while CT scans indicated local soft tissue swelling and flaky low-density shadows (Figures 4C–E). In cases where *M. tuberculosis* spread to adjacent bone tissue or throughout the body, MRI revealed multiple changes in bone and soft tissue, including decreased T1WI signal and increased T2WI signal (Figures 4F,G).

### 3.2 Comparison of pathological features

In the control group, all 59 cases (100%) were positive for molecular detection, and 37 cases (62.71%) were positive for acid-fast staining. In the CTB group, all 59 cases (100%) were positive for molecular detection, with a higher positivity rate of 41 cases (69.49%) for acid-fast staining. There was no statistical difference in the positivity rate for acid-fast staining and molecular detection between the two groups.

**Figure 5:**
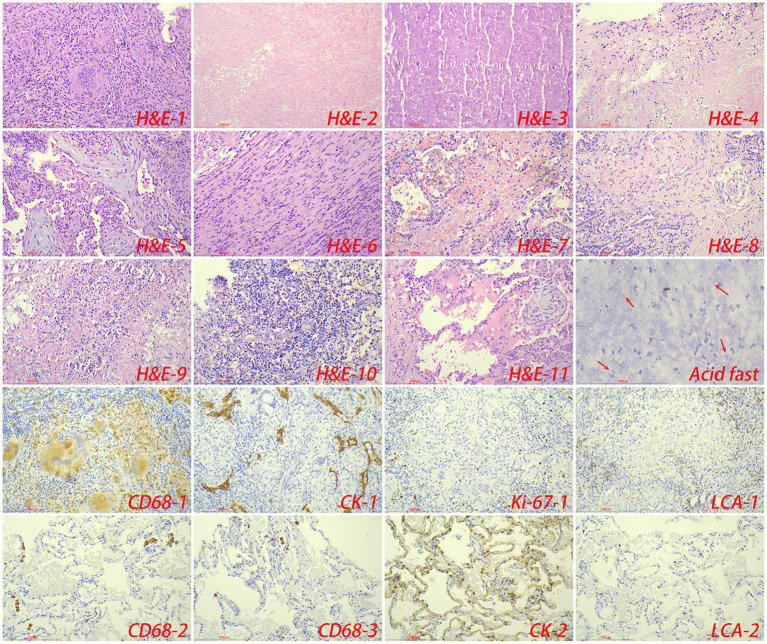
The typical pathological manifestations of pulmonary tuberculosis were chronic granulomatous inflammation (H&E-1) with caseous necrosis (H&E-2). H&E staining showed coagulation necrosis (H&E-3), inflammatory necrosis (H&E-4), mucous degeneration (H&E-5), fibroplasia (H&E-6), hemorrhage (H&E-7), acute inflammation (H&E-8), abscess (H&E-9), granulation tissue formation (H&E-10). Acid-fast staining showed *M. tuberculosis*. Immunohistochemical staining was used to label granuloma (CD68-1), alveolar epithelial cells (CK-1), proliferation rate (Ki-67-1) and lymphocyte infiltration (LCA-1) in tuberculosis lesions. Immunohistochemical staining was also used to label macrophage (CD68-2, CD68-3), alveolar epithelial cells (CK-2) and lymphocyte infiltration (LCA-2) in the surrounding normal lung tissue.

**Figure 6:**
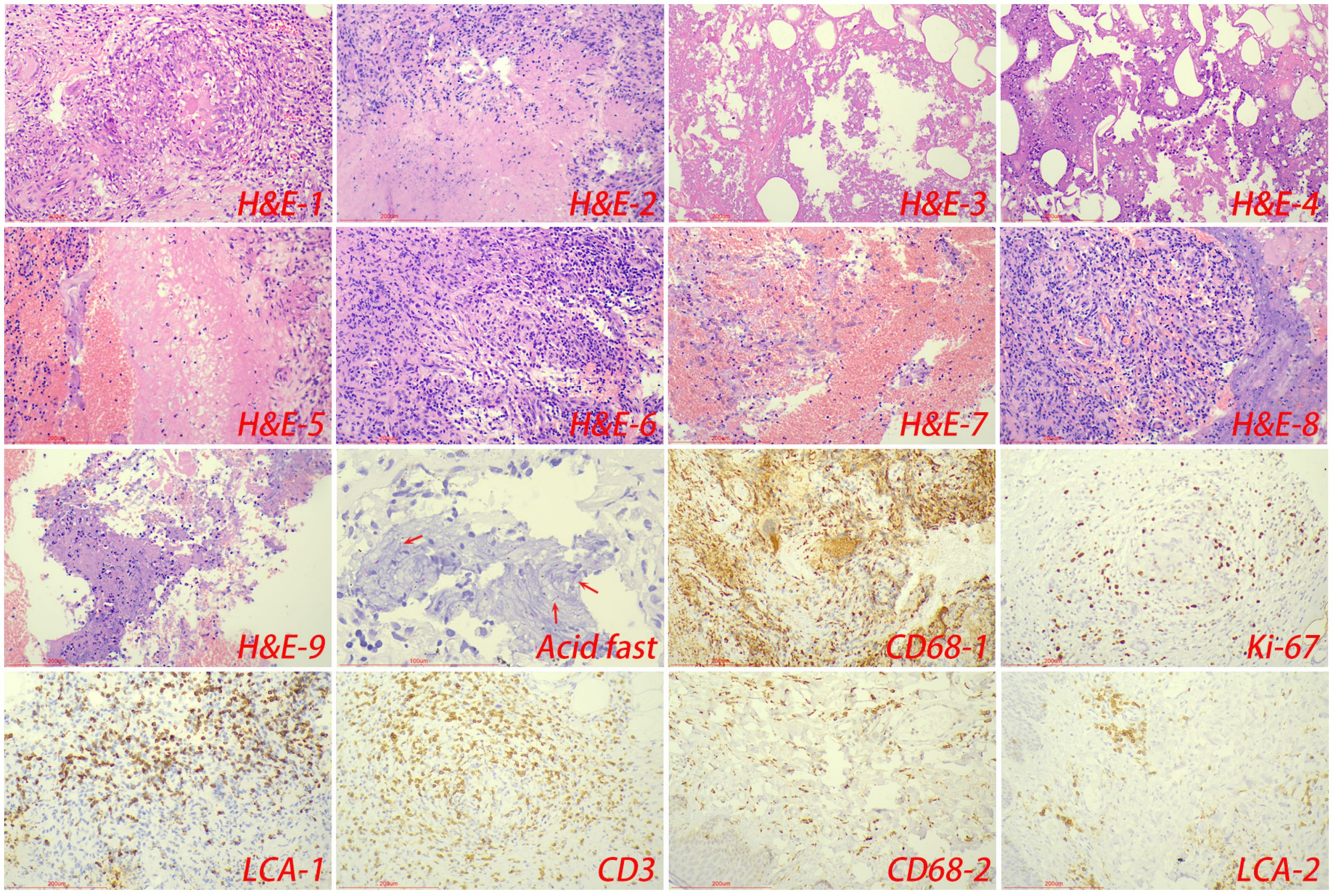
The typical pathological manifestations of CTB were chronic granulomatous inflammation (H&E-1) with caseous necrosis (H&E-2). H&E staining showed coagulation necrosis (H&E-3), inflammatory necrosis (H&E-4), exudation (H&E-5), fibroplasia (H&E-6), hemorrhage (H&E-7), granulation tissue formation (H&E-8), inflammatory necrosis (H&E-9). Acid-fast staining showed *M. tuberculosis*. Immunohistochemical staining was used to label granuloma (CD68-1), proliferation rate (Ki-67), lymphocyte (LCA-1) and T cell (CD3) infiltration in tuberculosis lesions. Immunohistochemical staining was also used to label macrophage (CD68-2), and lymphocyte infiltration (LCA-2) in the surrounding normal skin tissue.

Pathologically, chronic granulomatous inflammation was most common in the control group, observed in 37 cases (62.71%), followed by caseous necrosis in 21 cases (35.59%), coagulative necrosis in seven cases (11.86%), inflammatory necrosis in 21 cases (35.59%), acute inflammation in 19 cases (32.20%), hemorrhage in three cases (5.08%), fibroplasia in seven cases (11.86%), and exudation in five cases (8.47%). In the CTB group, chronic granulomatous inflammation was again most common, present in 50 cases (84.75%), with caseous necrosis in 15 cases (25.42%), coagulative necrosis in nine cases (15.25%), inflammatory necrosis in 17 cases (28.81%), acute inflammation in 12 cases (20.34%), hemorrhage in one case (1.69%), fibroplasia in seven cases (11.86%), and exudation in two cases (3.39%). Statistical analysis indicated a higher proportion of chronic granulomatous inflammation in the CTB group, but no significant differences in the proportions of other pathological features compared to the control group. There were no statistical differences in all pathological characteristics between secondary CTB and primary CTB. The results of immunohistochemistry indicated that the number of macrophages in the CTB group was over two times that of the control group, suggesting that macrophages play a significant role in the characteristic pathological changes associated with CTB. We also compared the number of macrophages and lymphocytes in normal tissues around the lesion and found that the number of macrophages and lymphocytes in the skin was significantly higher than that in the lung tissue (Figures 5, 6 and Table 1).

**Table 1:** Comparison of important pathological features between the control tuberculosis group and the CTB group.

Pathological features	Control (*n* = 59)	CTB (*n* = 59)	*χ*^2^ values	*p*-value
Chronic granulomatous inflammation	37 (62.71)	50 (84.75)	7.394	0.011
Caseous necrosis	21 (35.59)	15 (25.42)	1.439	0.318
Coagulative necrosis	7 (11.86)	9 (15.25)	0.289	0.789
Inflammatory necrosis	21 (35.59)	17 (28.81)	0.621	0.555
Inflammatory	19 (32.20)	12 (20.34)	2.144	0.209
Hemorrhage	3 (5.08)	1 (1.69)	1.035	0.619
Fibroplasia	7 (11.86)	7 (11.86)	0.000	1.000
Exudation	5 (8.47)	2 (3.39)	1.367	0.439
Acid-fast staining	37 (62.71)	41 (69.49)	0.605	0.560
Molecular detection	59 (100)	59 (100)	/	/
Ulceration	1 (1.69)	18 (30.51)	18.130	<0.001
No tuberculosis at other sites	46 (77.97)	26 (44.07)	14.251	<0.001
A history of tuberculosis at other sites	13 (22.03)	33 (55.93)	14.251	<0.001

The value outside the parentheses represents “number of patients,” while the value inside the parentheses represents “proportion (%).”

### 3.3 Comparison of drug resistance

Clinically, skin biopsy is easier and less risky than lung puncture. Therefore, we compared the drug resistance characteristics to determine whether the drug resistance of secondary CTB can guide the treatment of PTB. In the control group, seven cases (11.86%) were sensitive to all four drugs tested, while 12 cases (20.34%) showed no expression of drug resistance. A total of 40 cases (67.80%) exhibited drug resistance to any drug, with monoresistance found in 13 cases (22.03%), predominantly against rifampicin (RFP) in nine cases. For multidrug resistance, 12 cases (20.34%) were resistant to both RFP and isoniazid (INH), and another 12 cases showed resistance to RFP, INH, and streptomycin (STR). Additionally, two cases (3.39%) displayed polyresistance, specifically to RFP and STR.

In the CTB group, only one case (1.69%) was sensitive to all four drugs, and a higher proportion of 36 cases (61.02%) showed no expression of drug resistance. Drug resistance to any drug was observed in 22 cases (37.29%), with monoresistance noted in eight cases (13.56%), primarily against INH. Among the multidrug resistance types, resistance to RFP and INH was the most common, observed in 11 cases. Only one case was found to be resistant to INH and STR, with no other types of polyresistance detected.

Statistical analysis revealed significant differences in drug resistance patterns between the CTB group and the control group. Compared to the control group, the CTB group had a higher proportion of cases with no expression of drug resistance and relatively lower proportions of drug resistance to any drug, RFP + INH + STR resistance, and RFP resistance. Additionally, when comparing the secondary CTB group to the control group, the only significant difference found was in resistance to RFP + INH + STR (Tables 2, 3). We further compared the differences between secondary CTB and primary CTB, and found that the incidence of INH resistance was higher in the secondary CTB group.

**Table 2:** Comparison of overall drug resistance between the control group and the CTB group.

Drug resistance type	Control (*n* = 59)	CTB (*n* = 59)	*χ*^2^ values	*p*-value
Fully sensitive	7 (11.86%)	1 (1.69%)	4.827	0.061
No expression of drug resistance	12 (20.34%)	36 (61.02%)	20.229	0.000
Arbitrary drug resistance	40 (67.80%)	22 (37.29%)	11.012	0.002
Monoresistance	13 (20.03%)	8 (13.56%)	1.448	0.336
RFP resistant	9 (15.25%)	2 (3.39%)	4.912	0.027
INH resistant	3 (5.08%)	6 (10.17%)	1.083	0.490
STR resistant	1 (1.69%)	0 (0.00)	1.009	1.000
EMB resistant	0 (0.00)	0 (0.00)	0.000	0.000
Multidrug resistance	24 (40.68%)	13 (22.03%)	4.764	0.046
Resistance to RFP + INH	12 (20.34%)	11 (18.64%)	0.054	1.000
Resistance to RFP + INH + STR	12 (20.34%)	2 (3.39%)	8.104	0.008
Resistance to RFP + INH + EMB	0 (0.00)	0 (0.00)	0.000	0.000
Resistance to RFP + INH + STR + EMB	0 (0.00)	0 (0.00)	0.000	0.000
Polyresistance	15 (25.42%)	1 (1.69%)	14.172	0.000
Resistance to RFP + STR	2 (3.39%)	0 (0.00)	2.034	0.496
Resistance to RFP + EMB	0 (0.00)	0 (0.00)	0.000	0.000
Resistance to RFP + STR + EMB	0 (0.00)	0 (0.00)	0.000	0.000
Resistance to INH + STR	0 (0.00)	1 (1.69%)	1.009	1.000
Resistance to INH + EMB	1 (1.69%)	0 (0.00)	1.009	1.000
Resistance to INH + STR + EMB	0 (0.00)	0 (0.00)	0.000	0.000
Resistance to STR + EMB	0 (0.00)	0 (0.00)	0.000	0.000

RFP, rifampicin; INH, isoniazid; STR, streptomycin; EMB, ethambutol. The value outside the parentheses represents “number of patients,” while the value inside the parentheses represents “proportion (%).”

**Table 3:** Comparison of overall drug resistance between the control group and the secondary CTB group.

Drug resistance type	Control (*n* = 59)	Secondary CTB (*n* = 33)	*χ*^2^ values	*p*-value
Fully sensitive	7 (11.86%)	1 (3.03%)	2.080	0.251
No expression of drug resistance	12 (20.34%)	15 (45.45%)	6.438	0.017
Arbitrary drug resistance	40 (67.80%)	17 (51.52%)	2.380	0.179
Monoresistance	13 (20.03%)	8 (13.56%)	1.448	0.336
RFP resistant	9 (15.25%)	1 (3.03%)	3.264	0.089
INH resistant	3 (5.08%)	6 (18.18%)	4.113	0.065
STR resistant	1 (1.69%)	0 (0.00)	0.565	1.000
EMB resistant	0 (0.00)	0 (0.00)	0.000	0.000
Multidrug resistance	24 (40.68%)	9 (27.27%)	3.84	0.082
Resistance to RFP + INH	12 (20.34%)	8 (24.24%)	0.190	0.793
Resistance to RFP + INH + STR	12 (20.34%)	1 (3.03%)	5.225	0.028
Resistance to RFP + INH + EMB	0 (0.00)	0 (0.00)	0.000	0.000
Resistance to RFP + INH + STR + EMB	0 (0.00)	0 (0.00)	0.000	0.000
Polyresistance	15 (25.42%)	1 (1.69%)	14.172	0.000
Resistance to RFP + STR	2 (3.39%)	0 (0.00%)	1.144	0.535
Resistance to RFP + EMB	0 (0.00)	0 (0.00)	0.000	0.000
Resistance to RFP + STR + EMB	0 (0.00)	0 (0.00)	0.000	0.000
Resistance to INH + STR	0 (0.00)	1 (3.03%)	1.808	0.359
Resistance to INH + EMB	1 (1.69%)	0 (0.00)	0.565	1.000
Resistance to INH + STR + EMB	0 (0.00)	0 (0.00)	0.000	0.000
Resistance to STR + EMB	0 (0.00)	0 (0.00)	0.000	0.000

RFP, rifampicin; INH, isoniazid; STR, streptomycin; EMB, ethambutol. The value outside the parentheses represents “number of patients,” while the value inside the parentheses represents “proportion (%).”

### 3.4 Comparison of drug resistance gene mutations

In the control group, mutations in the rifampicin (RFP) resistance gene were observed in 35 cases (59.32%). The D516V mutation (aspartic acid to valine) was present in seven cases (11.86%), and single codon mutations including D516V, D516G, H526Y, H526D, and S531L were found in another seven cases (11.86%). Isoniazid (INH) resistance, detected in 28 cases (47.46%), frequently involved the -15M mutation. Streptomycin (STR) resistance was noted in 15 cases (25.42%), predominantly due to the 88M gene mutation. Ethambutol (EMB) resistance was characterized by the 306M2 mutation.

In the CTB group, there were 15 cases (25.42%) with RFP resistance gene mutations, primarily the D516V mutation. The distribution of other RFP resistance gene mutations was less pronounced. INH resistance was found in 20 cases (33.90%), all with the -15M mutation. STR resistance gene mutations were present in three cases (5.08%), with one case (1.69%) exhibiting the 88M gene mutation.

Among the 17 rpoB gene mutation classifications, the PTB control group had a significantly higher proportion of gene mutations compared to the CTB group. Notably, the D516V + D516G + H526Y + H526D + S531L mutation was present in 11.86% of the PTB control group but absent in the CTB group. The prevalence of STR resistance mutations and the 88M gene mutation was also higher in the PTB group compared to the CTB group. Further comparison between the secondary cutaneous TB group and the PTB control group revealed differences in RFP resistance gene mutations, D516V + D516G + H526Y + H526D + S531L site mutations, and STR resistance gene mutations (Tables 4, 5).

**Table 4:** Comparison of drug-resistant mutation sites between the control group and the CTB group.

Mutational site	Control (*n* = 59)	CTB (*n* = 59)	*χ*^2^ values	*p*-value
RFP	35 (59.32%)	15 (25.42%)	13.882	0.000
*D516V*	7 (11.86%)	8 (13.56%)	0.076	1.000
*D516G*	1 (1.69%)	0 (0.00)	1.009	1.000
*H526Y*	0 (0.00)	0 (0.00)	0.000	0.000
*H526D*	4 (6.78%)	0 (0.00)	4.140	0.119
*S531L*	1 (1.69%)	0 (0.00)	1.009	1.000
*D516V + D516G*	0 (0.00)	0 (0.00)	0.000	0.000
*D516V + D516G + H526Y*	0 (0.00)	0 (0.00)	0.000	0.000
*D516V + D516G + H526D*	2 (3.39%)	1 (1.69%)	0.342	1.000
*D516V + D516G + H526Y + H526D*	1 (1.69%)	1 (1.69%)	0.000	1.000
*D516V + D516G + H526Y + H526D + S531L*	7 (11.86%)	0 (0.00)	7.441	0.013
*D516V + H526D*	1 (1.69%)	3 (5.08%)	1.035	0.619
*D516V + H526Y*	0 (0.00)	1 (1.69%)	1.009	1.000
*D516V + H526Y + H526D*	3 (5.08%)	1 (1.69%)	1.035	0.619
*D516V + H526Y + H526D + S531L*	3 (5.08%)	0 (0.00)	3.078	0.244
*D516G + H526Y*	1 (1.69%)	0 (0.00)	1.009	1.000
*H526Y + H526D*	3 (5.08%)	0 (0.00)	3.078	0.244
*H526D + S531L*	1 (1.69%)	0 (0.00)	1.009	1.000
INH	28 (47.46%)	20 (33.90%)	2.248	0.189
*−15M*	23 (38.98%)	20 (33.90%)	0.329	0.702
*315M*	5 (8.47%)	0 (0.00)	5.221	0.057
*−15M + 315M*	0 (0.00)	0 (0.00)	0.000	0.000
STR	15 (25.42%)	3 (5.08%)	9.440	0.004
*43M*	6 (10.17%)	2 (3.39%)	2.145	0.272
*88M*	9 (15.25%)	1 (1.69%)	6.993	0.017
*43M + 88M*	0 (0.00)	0 (0.00)	0.000	0.000
EMB	1 (1.69%)	0 (0.00)	1.009	1.000
*306M2*	1 (1.69%)	0 (0.00)	1.009	1.000

RFP, rifampicin; INH, isoniazid; STR, streptomycin; EMB, ethambutol; D, aspartate; V, valine; G, glycine; H, histidine; Y, tyrosine; S, serine; L, leucine; W, tryptophan. The value outside the parentheses represents “number of patients,” while the value inside the parentheses represents “proportion (%).”

**Table 5:** Comparison of drug-resistant mutation sites between the control group and the secondary CTB group.

Mutational site	Control (*n* = 59)	Secondary CTB (*n* = 33)	*χ*^2^ values	*p*-value
RFP	35 (59.32%)	10 (30.30%)	7.132	0.009
*D516V*	7 (11.86%)	5 (15.15%)	0.202	0.750
*D516G*	1 (1.69%)	0 (0.00)	0.565	1.000
*H526Y*	0 (0.00)	0 (0.00)	0.000	0.000
*H526D*	4 (6.78%)	0 (0.00)	2.339	0.293
*S531L*	1 (1.69%)	0 (0.00)	0.565	1.000
*D516V + D516G*	0 (0.00)	0 (0.00)	0.000	0.000
*D516V + D516G + H526Y*	0 (0.00)	0 (0.00)	0.000	0.000
*D516V + D516G + H526D*	2 (3.39%)	1 (3.03%)	0.009	1.000
*D516V + D516G + H526Y + H526D*	1 (1.69%)	1 (3.03%)	0.177	1.000
*D516V + D516G + H526Y + H526D + S531L*	7 (11.86%)	0 (0.00)	4.238	0.047
*D516V + H526D*	1 (1.69%)	2 (6.06%)	1.279	0.292
*D516V + H526Y*	0 (0.00)	1 (3.03%)	1.808	0.359
*D516V + H526Y + H526D*	3 (5.08%)	0 (0.00)	1.735	0.550
*D516V + H526Y + H526D + S531L*	3 (5.08%)	0 (0.00)	1.735	0.550
*D516G + H526Y*	1 (1.69%)	0 (0.00)	0.565	1.000
*H526Y + H526D*	3 (5.08%)	0 (0.00)	1.735	0.550
*H526D + S531L*	1 (1.69%)	0 (0.00)	0.565	1.000
INH	28 (47.46%)	16 (48.48%)	0.009	1.000
*-15M*	23 (38.98%)	16 (48.48%)	0.782	0.389
*315M*	5 (8.47%)	0 (0.00)	2.957	0.156
*-15M + 315M*	0 (0.00)	0 (0.00)	0.000	0.000
STR	15 (25.42%)	2 (6.06%)	5.267	0.025
*43M*	6 (10.17%)	1 (3.03%)	1.534	0.415
*88M*	9 (15.25%)	1 (3.03%)	3.264	0.089
*43M + 88M*	0 (0.00)	0 (0.00)	0.000	0.000
EMB	1 (1.69%)	0 (0.00)	0.565	1.000
*306M2*	1 (1.69%)	0 (0.00)	0.565	1.000

RFP, rifampicin; INH, isoniazid; STR, streptomycin; EMB, ethambutol; D, aspartate; V, valine; G, glycine; H, histidine; Y, tyrosine; S, serine; L, leucine; W, tryptophan. The value outside the parentheses represents “number of patients,” while the value inside the parentheses represents “proportion (%).”

## 4 Discussion

CTB, as a rare form of extrapulmonary tuberculosis, has seen a steady increase in its infection rate worldwide, garnering increasing attention from clinicians (Gurel et al., 2022; Srihari et al., 2022). Clinically, CTB often presents as ulceration and subcutaneous nodules, which can be challenging to distinguish from other skin diseases (Kong et al., 2022; Mannava et al., 2022). This difficulty in differential diagnosis often leads to delayed identification, resulting in significant patient distress and potentially severe consequences. Pathology is an important diagnostic criterion for CTB, which is mainly characterized by granulomatous inflammation and caseous necrosis (Yadav, 2022). The positive rate of acid-fast staining is higher in the CTB group, possibly due to the larger volume of biopsy specimens. The advancement of molecular biology techniques has significantly bolstered the role of molecular detection in diagnosing and differentiating tuberculosis. Our study indicates a high positive rate of molecular detection in both pulmonary and cutaneous tuberculosis cases. Additionally, molecular testing is crucial for differentiating CTB from infections caused by non-tuberculous mycobacteria, such as *M. avium* and *M. gordonae*. Consequently, for cases involving ulcers that are non-responsive to long-term treatment or have difficulty healing, we recommend timely skin biopsies and pathological examinations to establish a definitive diagnosis.

Numerous studies have underscored granulomatosis as a pivotal aspect of the immune pathogenesis in *M. tuberculosis* infection. The formation of granulomas, which are primarily composed of macrophages, is believed to be a response to the activation of the immune system upon infection (Haddad et al., 2022). These macrophage-rich nodules act as a barrier, restricting the movement and proliferation of tuberculosis bacteria. Our study corroborates this understanding, showing that chronic granulomatous inflammation is a predominant pathological feature in both CTB and PTB. Upon comparing the pathological characteristics of CTB and PTB, we observed a relatively higher prevalence of granulomatous inflammation in CTB. However, other features such as caseous necrosis, coagulative necrosis, inflammatory necrosis, acute inflammation, hemorrhage, fibroplasia, and exudation did not exhibit significant differences between the two groups. This finding suggests that the variance in macrophage quantity might be a key factor influencing the distinct pathological features of CTB and PTB. Single-cell sequencing has revealed that macrophages, also known as Langhans giant cells, are the most abundant immune cells in the skin. These cells play a crucial role in maintaining the stability of the body’s immune barrier against external factors (Kolter et al., 2019). The high concentration of macrophages in the skin could facilitate the effective containment and destruction of *M. tuberculosis* upon its invasion, which might explain the significantly lower incidence of CTB compared to PTB. This observation highlights the importance of understanding the immune microenvironment in the skin and its role in the pathogenesis and clinical presentation of CTB. The lower incidence of CTB compared to other extrapulmonary tuberculosis forms may be partly attributed to the robust immune response in the skin, particularly the high concentration of macrophages. These immune cells become more active during infection, leading to a higher proportion of granulomatous inflammation. This response is especially pronounced when the immune system is compromised or when the bacterial load is excessive. Many cytokines play a crucial role in the formation of granulomas, including TNF-α and IFN-γ, and some immunosuppressive molecules may also be important mechanisms (Li et al., 2024).

Tuberculosis therapy, the rising incidence of drug-resistant tuberculosis poses a significant challenge. Many patients continue to experience inadequate responses or clinical deterioration, such as new lesions, persistent fever, weight loss, or relapse, even after prolonged standard anti-TB therapy (Ramesh et al., 2022; Rustad et al., 2022; Singal et al., 2022). Our study found a notable difference in the classification of “no expression of drug resistance” between the CTB group and the pulmonary tuberculosis (PTB) control group. This category, characterized by no results in drug resistance determination areas or incomplete color development, may be due to a relatively low bacterial load in CTB samples, reducing the sensitivity and specificity of detection methods. In comparing drug resistance in secondary CTB with the PTB control group, a significant difference was observed only in resistance to RFP + INH + STR. This finding suggests that secondary CTB foci may reflect the drug resistance profile of primary foci to some extent. D516V is located in the rifampicin resistance determinant region of the rpoB gene, which encodes the active center of the β subunit of RNA polymerase (Kabir et al., 2021). RFP is the core drug in the treatment regimen for CTB, and its resistance significantly increases the difficulty of treatment and the risk of failure (Bhandari et al., 2022). Given the convenience and lower invasiveness of skin biopsies compared to lung punctures, skin biopsy in cases of concurrent pulmonary and cutaneous tuberculosis can provide valuable guidance for clinical medication. It is worth noting that lung biopsy is primarily performed in cases where clinical diagnosis is unclear, to differentiate between malignant tumors and infectious lesions in the lungs. Clinically, the use of respiratory samples (sputum, etc.) for *M. tuberculosis* culture and molecular detection is a more commonly used and recommended method.

## 5 Conclusion

In summary, CTB is characterized by a higher proportion of granulomatous lesions, and the high number of macrophages in the skin may be an important reason. The similarity in drug resistance profiles between secondary CTB and PTB offers a potential avenue for guiding personalized anti-tuberculosis treatment. Skin biopsy in cases of concurrent pulmonary and cutaneous tuberculosis can provide valuable guidance for clinical medication. Our study thus contributes significant data for clinical diagnosis and the development of tailored treatment strategies.

## Data Availability

The original contributions presented in the study are included in the article/supplementary material, further inquiries can be directed to the corresponding authors.
